# A lightweight detector with hybrid pooling and checkerboard attention for solar panel anomalies

**DOI:** 10.1016/j.isci.2026.115106

**Published:** 2026-02-20

**Authors:** Xing Yang, Hongye Fang, Fan Yang, Kailiang Li, Ru Han, Tongjie Li

**Affiliations:** 1 College of Intelligent Manufacturing, Anhui Science and Technology University, Chuzhou 233100, China; 2 School of Electrical Engineering and Automation, Jiangsu Normal University, Xuzhou 221116, China; 3 College of Smart Agriculture, Nanjing Agricultural University, Nanjing 210031, China

**Keywords:** applied computing, applied computing in engineering

## Abstract

The reliable operation of solar-powered agricultural Internet of Things (IoT) devices heavily depends on the integrity of solar panels. However, monitoring these distributed assets for subtle anomalies such as bird droppings, cracks, and dust accumulation remains challenging under edge computational constraints. This paper presents YOLOv11-HPC, an optimized lightweight detector that incorporates a Hybrid Pooling Spatial Pyramid Pooling Fast module and a Dual-path Multi-scale Checkerboard Attention module. These components collectively improve multi-scale feature representation and introduce sparse attention-guided refinement, enabling accurate identification of small and complex anomalies with low computational overhead. Evaluated on a dedicated solar panel anomaly dataset, YOLOv11-HPC achieves an *mAP*_50_ of 84.1% and a precision of 94.13%, surpassing existing YOLO models and classical detectors. When deployed on an NVIDIA Jetson Orin NX, the model sustains real-time inference at over 55 FPS in FP16 format, confirming its practical suitability for edge-based agricultural IoT device monitoring and sustainable agricultural applications.

## Highlights

• Propose a solar panel anomaly detection method for agricultural IoT
• Enhance multi-scale small target feature representation
• Demonstrate practicality by comprehensive validation on the edge device

## Graphical abstract

**Figure:**
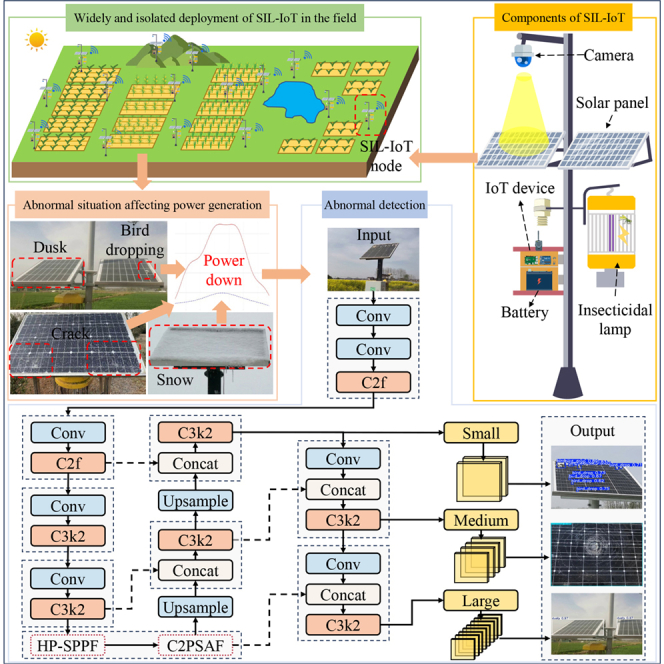

## Introduction

The global transition to sustainable energy has positioned photovoltaic (PV) technology as a cornerstone of renewable power generation, with rapidly expanding installed capacity worldwide.^1^^,^^2^ However, maintaining PV system efficiency over operational lifespans presents persistent challenges.^3^ Environmental factors including dust accumulation, bird droppings, and physical damage can cause significant power loss, hotspot formation, and permanent module degradation, directly undermining the economic viability and sustainability objectives of PV deployments.^4^

**Figure 1:**
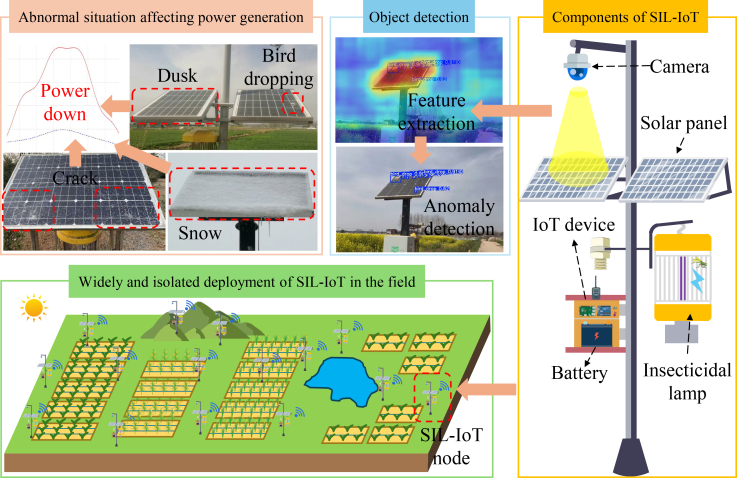
The composition structure and deployment status of the SIL-IoT, where a camera is used to detect anomalies of SIL-IoT solar panels for preventing decrease in power generation

These challenges are particularly critical in emerging agricultural applications such as solar insecticidal lamp Internet of Things (SIL-IoT) systems.^5^ As illustrated in Figure 1, these devices combine green energy with precision agriculture, using solar-charged batteries to power pest control operations while reducing chemical pesticide reliance.^6^ Their proliferation creates distributed micro-generation networks where individual unit reliability is essential. Failure not only disables the device but compromises localized pest management, potentially causing agricultural losses.^5^

The SIL-IoT operational environment imposes unique constraints: devices function as isolated nodes across remote fields without grid connection or routine maintenance access.^7^ Panels face exacerbated contamination from dust, pollen, and bird droppings, which was also amplified by the pest-attraction function.^8^ Crucially, even single-day charging interruption disrupts the pest-control cycle, making manual inspection of distributed units economically prohibitive. Automated monitoring thus becomes imperative for technological scalability.

Computer vision, particularly deep learning-based detection, offers a promising non-contact solution. While YOLO series models have advanced real-time object detection,^9^ their direct application to SIL-IoT anomaly detection faces domain-specific obstacles: (1) critical anomalies like bird droppings manifest as minimal-scale objects where standard detectors lose fine-grained features; (2) complex agricultural backgrounds trigger false positives; and (3) edge deployment necessitates lightweight models for onboard processing without cloud dependency.

This reveals a significant research gap in specialized visual detectors accommodating both the unique anomaly profile and operational constraints of agricultural Internet of Things (IoT) solar panels. To address this, we propose an optimized YOLO-based framework with three core contributions.

• A dedicated detection algorithm targeting agricultural PV anomalies, complemented by a real-world field dataset emphasizing small-object defects.
• An optimized attention mechanism enhancing focus on subtle anomalies while suppressing background interference.
• Comprehensive lightweight design ensuring edge deployment capability through architectural optimization and model compression.

The remainder of this article is organized as follows. Related work section provides a review of related work, covering existing research on PV panel anomaly detection and advancements in YOLO algorithms. STAR Methods section details the methodology, including the architecture of our proposed optimized model and the design of the proposed attention module. Results and discussion section presents the experimental setup, results, and a comprehensive discussion comparing the performance of our model against other benchmarks. Finally, conclusion section concludes the paper by summarizing our findings and outlining directions for future research.

### Related work

#### Anomaly detection in solar panels

Maintaining peak efficiency in PV systems necessitates robust anomaly detection. Existing methods fall into two main categories: electrical-based and vision-based techniques.

Electrical-based methods analyze current-voltage (I-V) and power-voltage (P-V) characteristics for fault detection.^10^ Techniques range from measuring fill factors to employing neural networks for output prediction, effectively identifying issues like short circuits and partial shading.^11^ While sensitive to electrical deviations, these approaches require specialized sensors integrated into circuits, making them cost-prohibitive for distributed IoT deployments like SIL-IoT. Moreover, they detect performance degradation without visually identifying anomaly types or locations.

Vision-based methods provide a non-contact alternative through image analysis. Early thermographic techniques using infrared cameras identified heat spots from cracks or shading,^12^ but high hardware costs limited applicability. Subsequent approaches used RGB cameras with traditional image processing (histogram thresholding and morphological operations), yet these remained sensitive to environmental conditions and required handcrafted features.^13^

Deep learning has transformed this field, with convolutional neural networks (CNNs) enabling accurate classification and semantic segmentation of anomalies.^14^ However, their computational intensity, particularly for segmentation, challenges real-time edge deployment. This creates an opportunity for efficient object detection models that balance accurate localization and classification with manageable computational costs.

More recently, the latter half of 2025 has seen a surge in deep learning-based solar fault detection models specifically optimized for edge deployment in drone-based and IoT inspection systems. These advancements prioritize high *mAP*_50_ while maintaining a lightweight computational profile. For example, the YOLOv8n-GBE model integrates Ghost convolutions and bi-directional feature pyramid network-efficient channel attention (BiFPN-ECA) to enhance localization precision with minimal overhead,^15^ while deep convolutional generative adversarial network (DCGAN)-driven data augmentation has been successfully applied to balance minority fault classes in lightweight YOLO frameworks.^16^

Furthermore, hardware-aware optimizations have become a critical focus. Architectures such as TinyTripleNet and pruned, low-rank optimized residual networks are specifically designed for Edge Tensor Processing Unit (TPU) acceleration.^17^^,^^18^ High-speed edge processing has also been achieved using field-programmable gate array (FPGA)-powered patch-wise reusable CNN intellectual property cores (IPs).^19^ Other innovative approaches, such as the AAPN-Tiny adaptive attention pyramid and hierarchical spatial feature extraction combined with gated recurrent unit (GRU) sequential modeling, have further improved multi-class fault diagnosis in constrained agricultural environments.^20^^,^^21^ Collectively, these works underscore the industry-wide transition toward structural efficiency and hardware-specific optimization, which directly informs the “precision-first” yet lightweight design of our proposed YOLOv11-HPC.

#### Evolution of object detection algorithms

The development of object detection algorithms provides the foundational context for our model selection, with three distinct paradigms emerging historically.

Traditional and two-stage detectors: pre-deep learning methods like Viola-Jones and HOG with support vector machine (SVM) classifiers relied on manual feature design, proving inadequate for complex multi-scale detection tasks.^22^ The deep learning revolution began with two-stage detectors, pioneered by the R-CNN family.^23^ Frameworks such as Faster R-CNN and Mask R-CNN first generate region proposals and then classify and refine them, achieving high localization accuracy at the cost of computational complexity and slow inference.^24^ This multi-stage processing makes them impractical for real-time edge applications.

One-stage detectors: one-stage detectors addressed speed limitations by directly predicting bounding boxes and class probabilities. The YOLO series,^25^ SSD,^26^ and RetinaNet^27^ represent this approach. YOLO’s regression-based formulation enables remarkable inference speeds while maintaining competitive accuracy. The architecture’s continuous evolution through versions v.3 to v.11, with improvements in backbone design, feature pyramid networks, and loss functions, demonstrates its consistent optimization for speed-accuracy balance, making it ideal for real-time applications.

Transformer-based detectors: inspired by natural language processing (NLP) successes, Vision Transformers introduced end-to-end detection paradigms. Detection transformer (DETR) eliminated hand-designed components like non-maximum suppression (NMS) through transformer encoder-decoder architectures and bipartite matching.^28^ While subsequent variants like Deformable DETR improved convergence and performance, transformer models generally suffer from computational intensity due to self-attention mechanisms. Their high memory demands and slower inference compared to CNN-based one-stage detectors limit suitability for resource-constrained edge deployment.

#### Motivation for selecting and optimizing YOLO

Our selection of YOLO as the foundational architecture stems from a systematic analysis of application-specific constraints in agricultural IoT environments.

The imperative for edge deployment necessitates a model capable of efficient operation on resource-constrained hardware. YOLO’s consistently demonstrated performance on edge devices with limited processing power, memory, and energy budgets makes it uniquely suitable.^29^ While detection accuracy remains crucial, the operational context of agricultural monitoring permits a different risk profile compared to safety-critical applications. Modern YOLO variants provide a robust accuracy baseline that can be strategically optimized for the specific anomaly “vocabulary” of PV panels without compromising edge compatibility.

The regression-based one-stage architecture inherently supports the high inference speeds required for continuous monitoring applications. Furthermore, YOLO’s modular design enables targeted enhancements through well-established optimization pathways. This flexibility allows integration of specialized components like lightweight attention mechanisms while maintaining the core efficiency framework and facilitates model compression through pruning and quantization for deployment.

Although transformer-based detectors achieve impressive accuracy and two-stage methods offer precise localization, their computational characteristics misalign with our edge-based requirements. YOLO presents the optimal foundation, balancing speed, accuracy, and modularity. Our work therefore builds upon this architecture, introducing refinements to address small-object detection limitations while preserving its inherent efficiency advantages.

#### Overview of YOLOv11-HPC architecture

The YOLOv11 architecture comprises three core components: backbone, neck, and detection head. The backbone extracts multi-scale features through convolutional layers and specialized modules including C3k2 (a CSPNet variant with bottleneck layers) and Spatial Pyramid Pooling Fast (SPPF), which employs successive 5 × 5 MaxPooling operations to efficiently expand the receptive field. The neck, typically implemented as a Path Aggregation Network (PANet), constructs a feature pyramid by fusing high-resolution shallow features with semantically rich deep features, enabling effective multi-scale detection. The detection head utilizes decoupled heads for classification and bounding box regression, generating predictions at three distinct scales (80 × 80, 40 × 40, and 20 × 20) optimized for small, medium, and large objects, respectively.

While demonstrating strong general detection performance, YOLOv11 shows limitations in solar panel anomaly detection for SIL-IoT applications. Specifically, it achieves high confidence on large anomalies like dust coverage but underperforms on subtle defects such as bird droppings and micro-cracks, where weak features are easily lost during network propagation. Additionally, edge deployment requirements demand both accuracy and computational efficiency.

**Figure 2:**
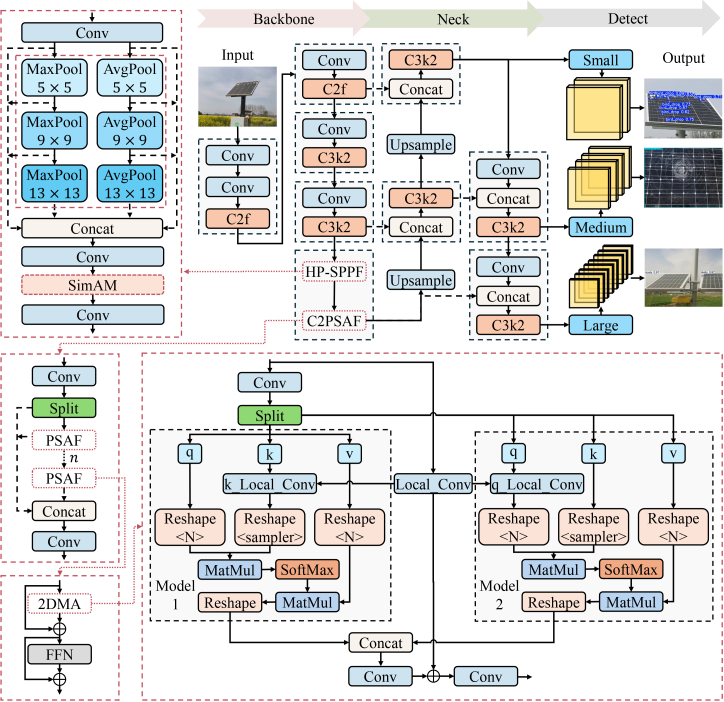
Architecture of the YOLOv11-HPC model for solar panel defect detection, where the small head with large feature maps (80 × 80) is specialized for detecting small objects (e.g., bird droppings), the medium head with medium feature maps (40 × 40) for medium objects (e.g., cracks or partial covering), and the large head with small feature maps (20 × 20) for large objects (e.g., large-area dust coverage) The proposed model incorporates an HP-SPPF module and a C2PSAF module within the backbone network to enhance feature extraction capability, particularly for micro-scale defects.

To address these challenges, we propose an optimized YOLOv11 architecture featuring targeted structural refinements to the backbone. While preserving the efficient one-stage detection paradigm of YOLO, our work introduces specialized architectural optimizations: (1) a Hybrid Pooling Spatial Pyramid Pooling Fast (HP-SPPF) module designed for high-fidelity feature preservation and (2) a CSP Structure with Dual-path Multi-scale Checkerboard Attention (C2PSAF) module. These components represent an intentional engineering evolution of the network to accommodate the subtle visual signatures of PV anomalies under edge constraints. The complete architecture is illustrated in Figure 2.

## Results

A comprehensive evaluation of the proposed solar panel anomaly detection method was conducted, assessing detection accuracy, computational efficiency, and generalization capability with emphasis on small target performance.

### Ablation experiment

A systematic ablation study evaluates the contributions of the HP-SPPF and C2PSAF modules, with results in Table 1. The baseline YOLOv11 provides a strong foundation. Variant A (C2PSAF only) improves overall *mAP* metrics and achieves the highest recall (85.41%), demonstrating enhanced anomaly sensitivity. Variant B (HP-SPPF only) achieves superior precision (92.53%) but suffers reduced recall, indicating a conservative detection tendency that avoids false positives at the cost of missed detections.

**Table 1:** Ablation experiment results of the proposed components

			*mAP*_50_	*mAP*_50–95_	Precision	Recall	F1-score	GFLOPs
Baseline	–	–	82.85	73.89	92.22	84.59	88.24	10.78
A	✓	–	83.78	75.01	91.9	**85.41**	**88.54**	10.88
B	–	✓	81.38	73.16	92.53	82.48	87.24	10.78
Proposed	✓	✓	**84.1**	**75.2**	**94.13**	82.99	88.2	10.88

Bold indicates the best performance of related metrics.

The complete proposed model achieves optimal performance, attaining the highest *mAP*_50_ (84.10%), *mAP*_50–95_ (75.20%), and precision (94.13%). This demonstrates synergistic integration: HP-SPPF provides enriched multi-scale features while C2PSAF enables intelligent refinement. The strategic trade-off of marginally reduced recall for substantially improved precision yields the most favorable balance for industrial anomaly detection.

To further evaluate the efficiency-accuracy trade-off of variant A and B, we conducted a quantitative analysis of its computational overhead in terms of giga floating-point operations per second (GFLOPs). As shown in Table 1, the integration of the C2PSAF module into the baseline YOLOv11 increases the computational cost from 10.78 GFLOPs to 10.88 GFLOPs (a marginal increment of only 0.10 GFLOPs or 0.9%). Compared to standard self-attention mechanisms, which exhibit quadratic complexity, our dual-path checkerboard sampling strategy effectively halves the sequence length, maintaining a computational profile comparable to the parameter-free SimAM module used in HP-SPPF (which maintains GFLOPs at 10.78). This minimal 0.9% increase in FLOPs yields a significant 1.25% improvement in *mAP*_50_ (from 82.85% to 84.10%), demonstrating the superior efficiency of the C2PSAF module in capturing long-range dependencies without the typical overhead of transformer-based components.

### Comparison with state-of-the-art methods

**Figure 3:**
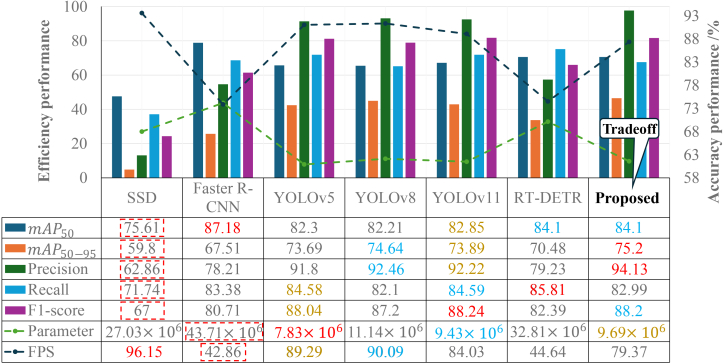
Comprehensive performance comparison of the proposed method with state-of-the-art detectors on the test set, where data are represented as mean The bar charts (left vertical axis) depict accuracy metrics. The line plots with dashed markers (right vertical axis) represent efficiency metrics. The numerical values for all metrics are provided in the table beneath the *x* axis, where the red font highlights the best-performing value for each metric, the blue font indicates the second-best value, and the brown font denotes the third-best value. The red dashed box is used to emphasize the worst-performing result for each metric.

Comprehensive comparison against established detectors (SSD, Faster R-CNN, YOLOv5/8/11-s variants, and RT-DETR-l) reveals the proposed model’s superior balance between accuracy and efficiency (Figure 3). Our method achieves leading precision (94.13%), exceeding the nearest competitor by 1.67%, which directly translates to reduced false alarms in operational settings. This advantage stems from the synergistic HP-SPPF and C2PSAF modules, which collectively enhance feature discrimination.

The experimental findings unequivocally demonstrate that the proposed model achieves a preeminent balance, excelling particularly in precision, which is a paramount metric for anomaly detection in industrial applications. In this context, a high precision is critical as it directly correlates with a low false alarm rate, preventing unnecessary and costly manual inspections triggered by erroneous alerts. Our model secures the highest precision of 94.13% among all evaluated methods. Notably, even compared to the Transformer-based RT-DETR, which manages a precision of only 79.23%, our model shows a significant reliability advantage for trustworthy automated inspection. This achievement is attributed to the synergistic effect of the HP-SPPF and C2PSAF modules, where the former provides a richer multi-scale foundation and the latter suppresses spurious background activations.

While the two-stage Faster R-CNN and the Transformer-based RT-DETR achieve high recall scores (83.38% and 85.81%, respectively), their precision is substantially lower than all YOLO-based models. RT-DETR achieves the highest recall among all benchmarks, suggesting high sensitivity to potential anomalies; however, this often comes at the cost of generating more false positives. For our application, the high precision of the proposed model is a more valuable attribute. Furthermore, our model ties with RT-DETR for the highest *mAP*_50_ (84.1%) and achieves the absolute highest *mAP*_50–95_ (75.2%), affirming its overall leading detection accuracy and robustness.

Regarding efficiency, the proposed model maintains a practical balance. Its parameter count (9.69 million) is significantly lower than that of RT-DETR (32.81 million) and Faster R-CNN (43.71 million), demonstrating the computational economy of our targeted optimizations. Although our inference speed (79.37 FPS) is lower than the early YOLO variants, it is nearly double that of RT-DETR (44.64 FPS) and far exceeds the 25 FPS operational threshold required for real-time monitoring. This strategic trade-off—sacrificing extreme raw speed for substantial gains in precision—makes the proposed model uniquely suitable for edge-based agricultural energy management, whereas RT-DETR’s high memory demand and lower FPS limit its practicality for resource-constrained SIL-IoT nodes.

### Generalization performance on open-source dataset

To further validate the robustness and generalization capability of the proposed YOLOv11-HPC beyond our specific SIL-IoT environment, we conducted additional experiments on a publicly available solar panel anomaly dataset, the Solarpanel-ggmtm.^30^ This dataset consists of 679 images captured under varied lighting and perspectives, significantly different from our agricultural field backgrounds.

As summarized in Table 2, the experimental results demonstrate that our proposed model maintains its competitive advantage in a cross-domain context. While the two-stage Faster R-CNN achieves a slightly higher *mAP*_50_ (45.46%), its precision is notably lower at 43.27%, leading to a high rate of false positives. In contrast, YOLOv11-HPC achieves the highest precision (65.60%) and F1-score (51.82%) among all evaluated models. Furthermore, compared to the baseline YOLOv11s, the proposed model shows consistent improvements across nearly all metrics, including a 0.19% gain in *mAP*_50_ and a substantial 10.49% leap in precision. This consistent performance across heterogeneous datasets proves that the HP-SPPF and C2PSAF modules provide generalized feature enhancement rather than being overfitted to a specific dataset.

**Table 2:** Quantitative performance comparison on the *Solarpanel-ggmtm* open-source dataset

	SSD	Faster R-CNN	YOLOv5	YOLOv8	YOLOv11	RT-DETR	Proposed
*mAP*_50_	31.30	**45.46**	35.59	41.92	42.63	42.92	42.82
*mAP*_50–95_	11.46	13.14	18.65	16.39	**20.88**	18.50	19.73
Precision	29.76	43.27	61.92	58.23	55.11	53.42	**65.60**
Recall	25.38	40.89	53.81	56.32	52.70	51.78	**57.36**
F1-score	30.51	44.34	45.20	48.75	48.08	47.60	**51.82**

Bold indicates the best performance of related metrics.

### Result visualization

**Figure 4:**
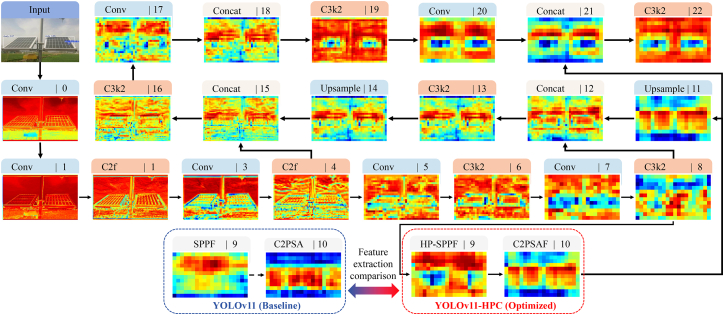
Visual analysis of feature map evolution across the YOLOv11-HPC architecture and a comparative study of feature extraction between the baseline (YOLOv11) and the optimized modules (HP-SPPF and C2PSAF)

To gain deeper insight into the internal workings of the proposed model and quantitatively validate its performance advantages, we present a detailed visual analysis of the feature maps generated at critical stages of the network. As illustrated in Figure 4, the evolution of feature representations throughout the entire YOLOv11-HPC architecture demonstrates a clear transition from low-level structural edges to high-level semantic abstractions.

Crucially, the “Feature extraction comparison” in Figure 4 highlights the efficacy of the proposed architectural enhancements. While the baseline YOLOv11 modules (SPPF and C2PSA) tend to generate relatively diffuse and noisy heatmaps, our optimized modules (HP-SPPF and C2PSAF) produce significantly more concentrated and semantically relevant activations. Specifically, the HP-SPPF module suppresses background clutter more effectively through its hybrid pooling strategy, and the C2PSAF module further refines the attention focus, ensuring that the network prioritizes genuine anomalous patterns over spurious environmental textures. This macroscopic enhancement across the network layers provides the foundation for the superior detection performance observed in specific anomaly categories discussed in the following paragraph.

**Figure 5:**
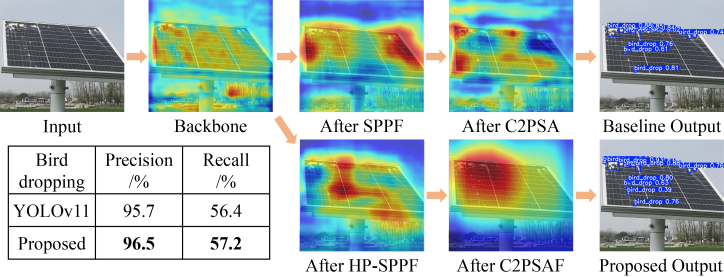
Comparative visualization of feature evolution and final detection for bird droppings, where data are represented as mean

Bird dropping (small object) detection: as illustrated in Figure 5, the input image containing small bird droppings presents a significant challenge. The feature maps from the initial backbone are diffuse and noisy, indicating a struggle to isolate the faint signatures of the small targets from the background. In the baseline YOLOv11, the SPPF module provides some initial feature concentration, but this focus is not sustained; the subsequent C2PSA module yields feature maps that are once again dispersed, failing to maintain a coherent representation of the anomalies. This visual finding correlates directly with the quantitative results, where the baseline achieves a recall of only 56.4%, meaning it misses a substantial portion of these subtle defects. In stark contrast, the proposed model demonstrates a markedly different and more effective feature evolution. The HP-SPPF module, with its hybrid multi-scale pooling, begins to enhance the relevant features. This process is decisively refined by the C2PSAF module, which produces intensely focused and localized activations precisely on the bird droppings. This superior feature refinement capability is quantified by the proposed model’s higher recall of 57.2% and the highest precision of 96.5%, confirming its enhanced ability to both find and correctly identify these elusive small anomalies with greater reliability.

**Figure 6:**
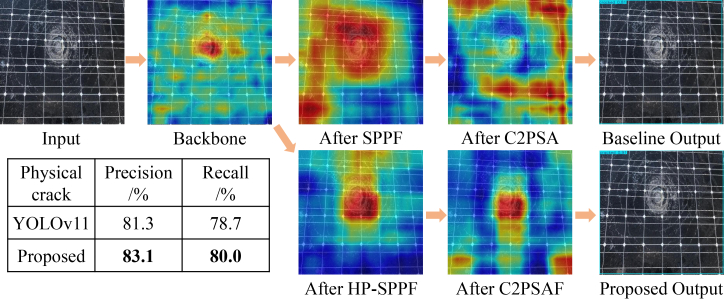
Comparative visualization of feature evolution and final detection for physical crack, where data are represented as mean

Physical crack (medium object) detection: the analysis of physical cracks, depicted in Figure 6, highlights the model’s ability to discriminate relevant features from distractors. The backbone already activates around the core crack regions but also exhibits significant erroneous activations in irrelevant areas. The baseline model’s SPPF module activates across the entire crack network but fails to suppress the background noise. Alarmingly, its C2PSA module then misdirects the focus entirely, leading to feature maps that do not correspond to the actual defect. This visual failure explains the baseline’s lower precision of 81.3% for this category. The proposed model, however, shows a controlled and progressive refinement. The HP-SPPF module effectively suppresses the spurious background activations observed in the backbone, directing attention toward the central crack regions. The C2PSAF module further sharpens this focus. While some minor erroneous focusing persists, the primary attention is correctly allocated. This superior discriminative ability is reflected in the higher quantitative performance, with the proposed model achieving a precision of 83.1% and a recall of 80.0%, outperforming the baseline on both fronts.

**Figure 7:**
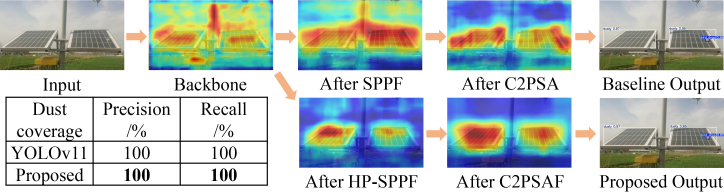
Comparative visualization of feature evolution and final detection for dust coverage, where data are represented as mean

Dust coverage (large object) detection: the visualization for large-area dust coverage, shown in Figure 7, reveals a distinct feature evolution pattern. The backbone features are characteristically scattered across the extensive anomalous region. The baseline model’s SPPF module shows limited improvement, producing features that remain somewhat unfocused. It is only after the C2PSA module that the baseline manages to consolidate the features reasonably well over the dusty panel. The proposed model exhibits an interesting and powerful dynamic: the HP-SPPF module alone does not show a strong focus, potentially due to the module’s role in capturing broad contextual information rather than immediate localization. However, the subsequent C2PSAF module performs exceptionally well, sharply focusing the activations to cover the dust-covered area effectively. Quantitatively, both models achieve perfect precision and recall (100%) for this obvious anomaly, but the feature maps demonstrate that the proposed model achieves this result through a more sophisticated and potentially more robust hierarchical feature refinement process.

In summary, the feature map visualizations, corroborated by category-specific metrics, provide compelling evidence for the efficacy of the proposed architectural enhancements. The HP-SPPF module serves as a powerful multi-scale context provider and noise suppressor, while the C2PSAF module acts as a dynamic and potent feature refiner. This synergy enables the proposed model to consistently evolve more discriminative and focused feature representations across different anomaly types and scales, directly translating into its superior quantitative detection performance, particularly for the challenging tasks of pinpointing small objects and accurately localizing medium-sized defects amid background clutter.

### Inference performance on edge device

Edge deployment capability was rigorously evaluated on NVIDIA Jetson Orin NX across multiple formats (.pt, ONNX, FP16, and INT8), with comprehensive results in Table 3. Our proposed model maintains its accuracy advantage in all formats, achieving the highest *mAP*_50_ (92.3% in .pt and 83.1% in INT8) and *mAP*_50–95_ (82.6% in .pt and 74.4% in INT8), demonstrating robust feature extraction capability even under severe quantization.

**Table 3:** Comprehensive edge device performance evaluation on NVIDIA Jetson Orin NX

Indicator	Format	YOLOv5	YOLOv8	YOLOv11	HP-SPPF	C2PSAF	Proposed
*mAP*_50_	PT	91.0	90.9	91.5	91.0	90.7	**92.3**
	ONNX	91.4	91.1	91.8	91.5	91.0	**92.3**
	FP16	90.2	90.2	90.9	91.0	87.8	**91.0**
	INT8	82.4	81.2	81.9	82.9	80.9	**83.1**
*mAP*_50–95_	PT	80.6	81.5	81.7	81.1	80.9	**82.6**
	ONNX	81.6	82.5	82.7	82.3	81.9	**82.6**
	FP16	80.6	81.3	81.2	81.4	81.2	**82.2**
	INT8	73.4	73.9	73.2	73.8	72.0	**74.4**
Precision	PT	91.3	90.1	93.0	93.0	**93.2**	91.4
	ONNX	91.4	90.3	93.2	93.2	**93.3**	91.3
	FP16	88.3	90.3	90.6	**92.5**	**92.5**	90.7
	INT8	87.8	88.0	92.4	**93.7**	86.5	90.7
Recall	PT	83.1	85.1	85.0	85.0	81.8	**86.7**
	ONNX	83.1	85.1	85.0	85.0	81.8	**86.7**
	FP16	83.6	82.6	83.7	84.6	80.0	**84.7**
	INT8	83.9	82.3	82.9	83.6	82.2	**84.0**
FPS	PT	**36.16**	34.41	31.16	29.04	28.34	25.59
	ONNX	**31.45**	25.70	25.11	19.28	28.07	17.67
	FP16	**69.11**	58.29	57.32	65.46	65.65	54.99
	INT8	61.78	**64.37**	57.50	55.64	50.65	49.05
Model size/MB	PT	**15.23**	21.48	18.30	18.30	18.79	18.79
	ONNX	**30.04**	42.66	36.16	36.16	37.96	37.96
	FP16	**18.21**	24.22	21.36	21.52	22.51	22.65
	INT8	**9.94**	12.79	12.30	12.37	13.35	13.52
GFLOPs	PT	**9.49**	14.33	10.78	10.78	10.88	10.88
	ONNX	**17.17**	25.92	19.24	19.24	19.43	19.43
	FP16	**8.58**	12.96	9.62	9.62	9.72	9.72
	INT8	**4.29**	6.48	4.81	4.81	4.86	4.8

Bold indicates the best performance of related metrics.

The computational-accuracy trade-off follows a clear pattern: FP16 emerges as the optimal balance, with our model achieving 91.0% *mAP*_50_ at 54.99 FPS, denoting a 115% speedup over .pt format with minimal accuracy loss. While INT8 quantization provides the smallest model size (13.52 MB) and significant speed improvement (49.05 FPS), it incurs substantial accuracy degradation (*mAP*_50_ drops to 83.1%), limiting its suitability for precision-critical applications.

In the context of agricultural IoT monitoring, the minimum real-time processing threshold is typically considered to be 25 FPS to ensure continuous and synchronized video analysis from edge-deployed cameras. Although our proposed model (79.37 FPS on PC and 54.99 FPS on edge) exhibits a slight reduction in inference speed compared to standard YOLOv11, it remains significantly above this operational requirement. This engineering trade-off is strategically prioritized because, in remote solar-powered deployments, the cost of false alarms (leading to unnecessary manual inspection) far outweighs the benefits of ultra-high frame rates. This precision-first engineering justification aligns with contemporary research on smart agriculture systems, which emphasizes that reliability and low false-positive rates are the primary prerequisites for autonomous field monitoring.^31^ The inference speeds of the proposed method are 25.59 FPS in .pt and 54.99 FPS in FP16, which remain well above real-time requirements for solar panel monitoring. The progressive performance scaling from .pt to FP16 to INT8 provides flexible deployment options, allowing system designers to prioritize either accuracy or efficiency based on specific application needs.

### Field deployment and robustness validation

**Figure 8:**
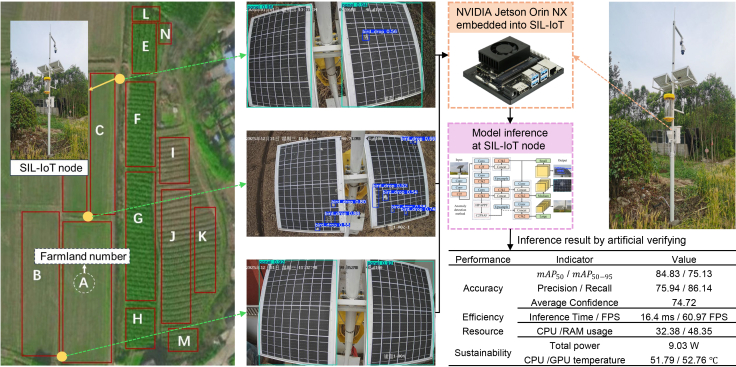
Real-world performance metrics of YOLOv11-HPC deployed on SIL-IoT nodes, where data are represented as mean

To verify the practical effectiveness and robustness of YOLOv11-HPC, we conducted on-site testing on three SIL-IoT nodes in Chuzhou, China. The testing period spanned from December 26, 2025, to January 3, 2026, involving 256 images captured in a real agricultural environment. Unlike the summer noon data used for training, these field tests covered diverse winter light conditions between 10:00 and 17:10. This setup accounts for lower solar elevations, seasonal shifts, and varied shadow patterns. The quantitative results of this deployment are summarized in Figure 8. The model maintained stable performance across these varying time frames, effectively identifying anomalies under challenging winter atmospheric conditions. It achieved an *mAP*_50_ of 84.83% and an average confidence of 74.72%. The inference process was highly efficient, with a speed of approximately 60.97 FPS. Furthermore, the hardware metrics confirmed high feasibility for remote deployment. The total power consumption was only 9.03 W, and the core temperatures remained stable near 52°C. These findings demonstrate the model’s reliability for year-round agricultural monitoring.

## Discussion

Experimental results and field testing establish our model as an effective solution for solar panel anomaly detection in IoT-edge scenarios. Under the hyperparameter configurations shown in Table 4, the model achieves an optimal balance between accuracy and deployment practicality. The method delivers state-of-the-art results and sets a precision benchmark of 94.13%. This high precision is critical for minimizing false alarms in remote operational environments. Such performance stems from the synergistic operation of the HP-SPPF and C2PSAF modules. These components provide multi-scale contextual foundations and enable dynamic feature refinement.

**Table 4:** Hyperparameters setting of the model training

Image	Batch size	Epoch	Optimizer	lr0	lrf	cos_lr	Seed
640 × 640	16	330	SGD	0.01	0.01	True	2025

The quantitative evaluation on the open-source dataset further highlights the distinctive behavior of our architecture compared to traditional detectors. From an engineering physics perspective, while two-stage models like Faster R-CNN often achieve high sensitivity through region proposal networks (RPNs), they are prone to over-triggering on background noise or structural textures that mimic anomalies, leading to a proliferation of false positives in lower quality datasets. In contrast, our YOLOv11-HPC employs a “precision-first” design. The C2PSAF module acts as a dynamic feature refiner that utilizes checkerboard-pattern sparse attention to suppress spurious background activations, while the HP-SPPF module preserves contextual information to avoid over-fitting on sharp, localized noise. This architectural strategy ensures that detection outputs are only generated when semantic features are sufficiently distinctive—a critical requirement for autonomous monitoring in remote environments where the cost of false alarms far outweighs the benefits of ultra-high frame rates.

The enhanced capability introduces a computational trade-off, with our architecture exhibiting the highest GFLOPs and lowest FPS among YOLO-family models on edge devices. However, this strategic compromise sacrifices marginal inference speed for a substantial improvement in detection reliability, achieving a precision of 94.13% while maintaining frame rates (e.g., 54.99 FPS on FP16) that far exceed the practical real-time requirement of 25 FPS. In addition, the field testing confirms that the system operates at a low power of 9.03 W and maintains thermal stability near 52°C. These metrics ensure that the system provides high-fidelity data without being over-engineered for raw speed.

### Conclusion

This study presents YOLOv11-HPC, a lightweight detector optimized for solar panel anomaly inspection in SIL-IoT systems. The architecture incorporates the HP-SPPF and C2PSAF modules to enhance multi-scale feature representation and enable efficient feature refinement. Experimental results demonstrate state-of-the-art performance, achieving an *mAP*_50_ of 84.1% and a precision of 94.13%. The robustness of the model is verified through evaluations on an open-source dataset and field deployment. During real-world testing, the model sustained a stable *mAP*_50_ of 84.83% and an inference speed of 60.97 FPS with a low power consumption of 9.03 W on edge hardware. These findings confirm the practical feasibility and reliability of the proposed method for long-term agricultural monitoring. Future research will focus on model compression for microcontrollers and temporal analysis for predictive maintenance. These directions will further enhance the intelligence and sustainability of agricultural energy management systems.

### Limitations of the study

Despite its high precision, the study possesses limitations in dataset diversity. The primary training images were acquired during summer months under relatively uniform illumination. This explains the higher baseline performance compared to open-source benchmarks. Lower overall accuracy on public datasets results from inherent quality and sample-size limitations. High sensor noise and low resolution increase the difficulty of feature convergence in those cases. However, our winter field tests from December 2025 to January 2026 demonstrate promising adaptability under low solar elevation. These tests address concerns regarding seasonal variations and illumination changes. Future research will focus on cross-domain data augmentation to further enhance the robustness of sustainable agricultural energy systems.

## Resource availability

### Lead contact

Further information and requests for resources should be directed to and will be fulfilled by the lead contact, Xing Yang (xingyang@ahstu.edu.cn).

### Materials availability

This study did not generate new materials.

### Data and code availability

• The image dataset has been deposited at Mendeley Data and is publicly available as of the date of publication. The website is listed in the key resources table.
• The code associated with this article has been deposited at GitHub and is publicly available as of the date of publication. The website is listed in the key resources table. https://github.com/harryyangx/Solar-Panel-Anomaly-Detection-Dataset-Based-on-Solar-Insecticidal-Lamp-Internet-of-Things.
• Any additional information required to reanalyze the date reported in this paper is available from the lead contact upon request.

## Acknowledgments

This research was supported by the 10.13039/501100001809National Natural Science Foundation of China under grant 62402003, the 10.13039/501100006247Anhui Science and Technology University Talent Introduction Project under grant RCYJ202402, and Natural Science Research Project of Anhui Educational Committee under grant 2023AH051856.

## Author contributions

X.Y.: writing – original draft, writing – review and editing, project administration, and methodology; H.F.: writing – original draft, data curation, and conceptualization; F.Y.: visualization and software; K.L.: resources and data curation; R.H.: validation and investigation; T.L.: supervision and resources. All authors have read and agreed to the published version of this article.

## Declaration of interests

The authors declare no competing interests.

## STAR★Methods

### Key resources table

**Table:** 

REAGENT or RESOURCE	SOURCE	IDENTIFIER
**Deposited data**		

Solarpanel-ggmtm^30^	Roboflow	https://universe.roboflow.com/solarpaneldataset/solarpanel-ggmtm
Solarpanels datasets^32^	This paper	https://doi.org/10.17632/8x7wjsr94v.1

**Software and algorithms**		

Python 3.11	Python Software Foundation	https://www.python.org
Anaconda 3	Anaconda, Inc.	https://www.anaconda.com
Pythoch 2.6	PyTorch Foundation	https://pytorch.org
YOLOv11-HPC	This paper	https://doi.org/10.5281/zenodo.18628189
YOLOv5s	Ultralytics	https://github.com/ultralytics/ultralytics/tree/main/ultralytics/cfg/models/v5
YOLOv8s	Ultralytics	https://github.com/ultralytics/ultralytics/tree/main/ultralytics/cfg/models/v8
YOLOv11s	Ultralytics	https://github.com/ultralytics/ultralytics/tree/main/ultralytics/cfg/models/11
RT-DETR-l	Zhao et al.^33^	https://doi.org/10.48550/arXiv.2304.08069
Faster R-CNN	Girshick Ross^34^	https://github.com/rbgirshick/fast-rcnn
SSD	Liu et al.^26^	https://github.com/weiliu89/caffe/tree/ssd
SDK Manager	Nvidia	https://developer.nvidia.com/sdk-manager

**Other**		

RTX4090(Training)	Nvidia	24GB
Jetson Orin NX	Nvidia	8GB

### Experimental model and study participant details

The study utilized a dataset of 3,000 images sourced from 20 SIL-IoT nodes deployed in Baguazhou, Nanjing, China (installed in December 2023). Data collection was conducted between April and July 2025, primarily during peak daylight hours (high noon) to ensure high-visibility capture of surface anomalies. Images were acquired using integrated node cameras and mobile devices from various overhead and oblique angles, providing a consistent view of bird droppings, cracks, and dust coverage. The dataset was split into training, validation, and test sets at an 80:10:10 ratio. While the initial training data was concentrated under optimal illumination, its real-world applicability was further validated through subsequent field testing.

Evaluation metrics included accuracy measures (*mAP*_50_, *mAP*_50-95_, precision, recall, F1-score) and efficiency indicators (parameters, FPS, model size, GFLOPs). All models were trained on an Ubuntu server with Intel I9-14900K CPU and NVIDIA RTX 4090 GPU using PyTorch 2.6, with inference evaluation conducted on NVIDIA Jetson Orin NX across multiple formats (.pt, ONNX, FP16, INT8) to assess edge deployment feasibility. Hyperparameter configurations are detailed in Table 4.

### Method details

#### HP-SPPF module

The standard Spatial Pyramid Pooling Fast (SPPF) module relies exclusively on MaxPooling, which often overlooks subtle texture patterns and average intensity variations.^35^^,^^36^ In solar panel inspection, defects exhibit diverse morphological characteristics. Bird droppings appear as high-frequency, high-contrast salient points. Cracks manifest as linear or irregular edges with specific orientations. Conversely, dust coverage presents as diffuse, low-contrast regions that blur global boundaries. To address these challenges, we propose the HP-SPPF module (Algorithm 1).

Algorithm 1 **HP-SPPF module**

**Table:** 

**Input:** Input feature map $X\in R^{C\times H\times W}$
**Output:** Output feature map $Y_{out}\in R^{C^{\prime }\times H\times W}$
begin
*X*′←*Conv*_1×1_(*X*); // Channel reduction
*Y*_*list*_←[*X*′]; // Initialize list with identity feature
for *k*∈{5,9,13} **do**
$Y_{\max}^{k}\leftarrow MaxPool_{k\times k}\left(X^{\prime }\right)$; // Max pooling path
$Y_{avg}^{k}\leftarrow AvgPool_{k\times k}\left(X^{\prime }\right)$; // Average pooling path
$Y_{list}.append\left(Y_{\max}^{k},Y_{avg}^{k}\right)$
**end**
*Y*_*concat*_←*Concat*(*Y*_*list*_); // Channel-wise concatenation
*Y*_*out*_←*Conv*_1×1_(*Y*_*concat*_); // Channel fusion and projection
**return***Y*_*out*_
**end**

Unlike the sequential design of SPPF, HP-SPPF introduces a dual-path aggregation strategy. Each branch integrates MaxPooling and Average Pooling to capture both salient transitions and regional distributions. For small-scale defects like bird droppings, the first pooling layer employs a 5×5 kernel. This small receptive field (*RF*) prevents the loss of micro-features. The MaxPooling operation is defined as:

(Equation 1) $$y_{\max}=\max_{i\in \Omega }\left(x_{i}\right)$$

where *x*_*i*_ denotes the pixel values within the local neighborhood Ω. This operation effectively preserves the high-bright “mutation” signals of bird droppings against the dark panel background.

For medium-scale features such as cracks, the module utilizes a cascaded structure with a 9×9 kernel. Since the stride *S*=1, the equivalent receptive field *RF*_*k*_ increases progressively:

(Equation 2) *RF*_*k*_=*RF*_*k*-1_+(*k*-1)

This allows the network to perceive the continuity and trend of cracks rather than isolated points. To detect large-area dust coverage, the third layer (13×13 kernel) achieves an extensive receptive field of 25. Here, Average Pooling is critical as it senses the “overall darkening” trend by calculating the regional mean:

(Equation 3) $$y_{avg}=\frac{\sum_{i\in \Omega }x_{i}}{\left|\Omega \right|}$$

where *y*_*avg*_ is the averaged output and |Ω| is the number of pixels in the window.

To suppress background noise, we incorporate the SimAM parameter-free attention mechanism. It calculates the “energy” of each neuron to identify importance:

(Equation 4) $$e_{t}\left(w_{t},b_{t},y,x_{i}\right)=\frac{1}{n-1}\sum_{i=1}^{n-1}{\left(-1-\left(w_{t}x_{i}+b_{t}\right)\right)}^{2}+{\left(1-\left(w_{t}t+b_{t}\right)\right)}^{2}$$

where *e*_*t*_ is the energy function for the target neuron *t*, *w*_*t*_ and *b*_*t*_ are the weight and bias transforms, and *x*_*i*_ are other neurons in the channel. Lower energy indicates higher distinctiveness against the background.

#### C2PSAF module

To capture long-range dependencies without excessive computational costs, we introduce the C2PSAF block, which integrates a Dual-path Multi-scale Checkerboard Attention (PSAF) module into a Cross Stage Partial (CSP) structure.^37^ This module addresses the difficulty of identifying cracks, which require both local edge information and global context.

The feature map is first split into two branches:

(Equation 5) *x*_1_,*x*_2_=Split(*F*_*in*_)

where *F*_*in*_ is the input, *x*_1_ is the identity branch, and *x*_2_ is the attention branch. Within the PSAF module (Algorithm 2), two asymmetric paths are utilized (Algorithm 3).

Algorithm 2 **PSAF module**

**Table:** 

**Input:** Input tokens F $\in R^{N\times C}$, Projection matrices *W*^*Q*^, *W*^*K*^, *W*^*V*^
**Output:** Refined tokens $O\in R^{N\times C}$
**begin**
*Q*,*K*,*V*←*FW*^*Q*^,*FW*^*K*^,*FW*^*V*^; // Linear projection // Path 1: Global Q @ Sampled Local K $\hat{K},\hat{V}\;\leftarrow SymmetricPadding\left(K,V\right)$ // Boundary Handling: Apply symmetric padding to K, V
$K_{ls},V_{ls}\leftarrow CheckerboardSample\left(\hat{K},\hat{V}\right)$; // Sparse sampling
${\hat{K}}_{ls}\leftarrow Conv1D\left(K_{ls}\right)$; // Local context enhancement
$A_{1}\leftarrow MatMul\left(Q,{\hat{K}}_{ls}^{T}\right)/\sqrt{d_{k}}$; // Scaled attention scores
*O*_1_←*MatMul*(*Softmax*(*A*_1_),*V*_*ls*_); // Weighted sum of sampled *V* // Path 2: Sampled Local Q @ Global K $\hat{Q}\;\leftarrow SymmetricPadding\left(Q\right)$ // Similarly for Path 2 with symmetric padding on Q
*Q*_*ls*_←*CheckerboardSample*(*Q*); // Sparse sampling (shifted pattern)
${\hat{Q}}_{ls}\leftarrow Conv1D\left(Q_{ls}\right)$; // Local context enhancement
$A_{2}\leftarrow MatMul\left({\hat{Q}}_{ls},K^{T}\right)/\sqrt{d_{k}}$; // Scaled attention scores
$O_{2\_sampled}\leftarrow MatMul\left(Softmax\left(A_{2}\right),V\right)$; // Output for sampled positions
$O_{2}\leftarrow Scatter\left(O_{2\_sampled}\right)$; // Map back to full sequence
*O*_*concat*_←*Concat*(*O*_1_,*O*_2_);
*O*←*Linear*(*O*_*concat*_); // Projection layer **return***O*
**end**

Algorithm 3 **C2PSAF module**

**Table:** 

**Input:** Input feature map $X_{in}\in R^{C\times H\times W}$
**Output:** Output feature map $Y_{out}\in R^{C\times H\times W}$
**begin**
*X*_0_,*X*_1_←*Split*(*X*_*in*_); // Split input into two parts
for *i*←1**to***n***do**
*X*_1_←*PSAFModule*(*X*_1_); // Process one part through *n* PSAF modules
**end**
*Y*_*concat*_←*Concat*(*X*_0_,*X*_1_); // Merge processed and identity paths
*Y*_*out*_←*Conv*(*Y*_*concat*_); // Final fusion convolution
**return***Y*_*out*_
**end**

Path 1 focuses on local details of small/medium targets (bird droppings, cracks) by applying global queries (*Q*) to sparsely sampled local keys (*K*):

(Equation 6) $$A_{1}=S\text{oftmax}\left(\frac{Q_{g}{\left(K_{local}\right)}^{T}}{\sqrt{d}}\right),{Output}_{1}=A_{1}V_{local}$$

where *A*_1_ is the attention score, *d* is the scaling factor, and *K*_*local*_, *V*_*local*_ are the sampled local key and value matrices.

Path 2 captures the global distribution of dust by using local queries against global keys:

(Equation 7) $$A_{2}=S\text{oftmax}\left(\frac{Q_{local}{\left(K_{g}\right)}^{T}}{\sqrt{d}}\right),{Output}_{2}=A_{2}V_{g}$$

where *Q*_*local*_ represents the sampled local query. To maintain continuity at the boundaries of the solar panel, we implement a symmetric padding strategy^38^:

(Equation 8) *P*_*sym*_(*x*)=[*x*_*flip*_,*x*,*x*_*flip*_]

where *x*_*flip*_ denotes the mirrored boundary pixels. This prevents edge artifacts and ensures a complete receptive field for small anomalies near the frame.

The features are further refined using a Feed-Forward Network (FFN) and Group Normalization (GN):

(Equation 9) *X*_*refined*_=GN(FFN(*X*_*attn*_)+*X*_*attn*_)

where *X*_*attn*_ is the output of the dual-path fusion. Finally, we apply a location encoding via grouped convolution to enhance spatial perception:

(Equation 10) *PE*(*X*)=DWConv(*X*)

(Equation 11) *F*_*out*_=Proj(*X*_*refined*_+*PE*(*X*))

where *PE* is the position encoding and DWConv is the depth-wise grouped convolution.

### Quantification and statistical analysis

Evaluation metrics in this study encompass precision, recall, F1-score, *mAP*_50_, *mAP*_50-95_, and FPS, to comprehensively assess the model's detection performance and practical applicability. Precision reflects the accuracy of the model by measuring the proportion of true positive anomalies among all predicted anomalies, thus reducing misclassification of normal solar panels as defective or dusty. Recall evaluates the completeness of detection by calculating the ratio of successfully identified true anomalies, which contributes to avoiding missing small targets such as bird droppings. The F1-score, as the harmonic average of precision and recall, provides a balanced indicator of both accuracy and coverage, making it suitable for scenarios where targets of different sizes are unevenly distributed in field environments. *mAP*_50_, with an IoU threshold of 0.5, calculates the mean average precision across all categories, offering a lenient yet effective metric for overall detection performance. In contrast, *mAP*_50-95_ extends the evaluation across ten IoU thresholds from 0.5 to 0.95, providing a robust measure of model consistency under varying strictness levels, particularly important for assessing precise boundary alignment in crack detection. FPS quantifies the inference speed in frames per second, directly indicating whether the model meets real-time monitoring requirements in practical applications. The specific formulas used for these metrics are as follows:

(Equation 12) $$\text{Precision}:P=\frac{\text{TP}}{\text{TP}+\text{FP}}*100\%$$

(Equation 13) $$\text{Recall}:R=\frac{\text{TP}}{\text{TP}+\text{FN}}*100\%$$

(Equation 14) $$F1-\text{score}=\frac{2\times P\times R}{P+R}*100\%$$

(Equation 15) $$AP=\int_{0}^{1}P\left(R\right)\;dR$$

(Equation 16) $${mAP}_{50}=\frac{\sum_{i=1}^{C}{AP}_{i,\text{IOU}=0.5}}{C}$$

(Equation 17) $${mAP}_{50-95}=\frac{\sum_{\tau =0.5}^{0.95}{mAP}_{\text{IOU}=\tau }}{10}$$

(Equation 18) $$\text{FPS}=\frac{1}{t}$$

where TP, FP, and FN denote true positives, false positives, and false negatives, respectively; *C* is the number of categories; and *t* represents the inference time per image.
